# Preformulation Characterization and Stability Assessments of Secretory IgA Monoclonal Antibodies as Potential Candidates for Passive Immunization by Oral Administration

**DOI:** 10.1016/j.xphs.2019.07.018

**Published:** 2020-01

**Authors:** Yue Hu, Ozan S. Kumru, Jian Xiong, Lorena R. Antunez, John Hickey, Yang Wang, Lisa Cavacini, Mark Klempner, Sangeeta B. Joshi, David B. Volkin

**Affiliations:** 1 Department of Pharmaceutical Chemistry, Vaccine Analytics and Formulation Center (VAFC), University of Kansas, Lawrence, Kansas 66047; 2 MassBiologics of the University of Massachusetts Medical School, Boston, Massachusetts 02126

**Keywords:** secretory immunoglobulin A, immunoglobulin G, enterotoxigenic *Escherichia coli*, physicochemical characterization, formulation, stability, oral delivery, immunization, ELISA, enzyme-linked immunosorbent assay, ETEC, enterotoxigenic *Escherichia coli*, Fab, antigen binding fragment, Fc, crystallizable fragment, GdnHCl, guanidine hydrochloride, HC, heavy chain, IgG, immunoglobulin G, LC, light chain, LT, heat labile enterotoxin, mAb, monoclonal antibody, MW, molecular weight, PEG, polyethylene glycol, PTM, post translational modifications, SC, secretory component, SDS-PAGE, sodium dodecyl sulfate–polyacrylamide gel electrophoresis, SE-HPLC, size exclusion high performance liquid chromatography, SGF, simulated gastric fluid, sIgA, secretory immunoglobulin A, SV-AUC, sedimentation velocity analytical ultracentrifugation

## Abstract

Enterotoxigenic *Escherichia coli* (ETEC) is a major cause of diarrheal disease among children in developing countries, and there are no licensed vaccines to protect against ETEC. Passive immunization by oral delivery of ETEC-specific secretory IgAs (sIgAs) could potentially provide an alternative approach for protection in targeted populations. In this study, a series of physiochemical techniques and an *in vitro* gastric digestion model were used to characterize and compare key structural attributes and stability profiles of 3 anti–heat-labile enterotoxin mAbs (sIgA1, sIgA2, and IgG1 produced in CHO cells). The mAbs were evaluated in terms of primary structure, N-linked glycan profiles, size and aggregate content, relative apparent solubility, conformational stability, and *in vitro* antigen binding. Compared to IgG1 mAb, sIgA1 and sIgA2 mAbs showed increased sample heterogeneity, especially in terms of N-glycan composition and the presence of higher molecular weight species. The sIgA mAbs showed overall better physical stability and were more resistant to loss of antigen binding activity during incubation at low pH, 37°C with pepsin. These results are discussed in terms of future challenges to design stable, low-cost formulations of sIgA mAbs as an oral supplement for passive immunization to protect against enteric diseases in the developing world.

## Introduction

Diarrheal diseases are the second leading cause of death in developing countries, especially in sub-Saharan Africa and South Asia,^1^, ^2^, ^3^ with ∼0.6 million children under 5 years of age dying each year due to complications caused by severe diarrhea.^4^, ^5^ A major cause of diarrhea is from drinking water contaminated by pathogenic bacteria, viruses, or parasites.^4^ Enterotoxigenic *Escherichia coli* (ETEC) is the most common bacterial cause of diarrhea-associated mortality, which leads to approximately one quarter of all diarrheal episodes for infants and children less than 5 years of age.^6^, ^7^, ^8^, ^9^ To further complicate these problems, enhanced antibiotic resistance has been found in many ETEC strains.^10^, ^11^, ^12^ Thus, the development of an ETEC vaccine is considered the most effective and feasible strategy to prevent diarrheal diseases among children in developing countries^13^, ^14^ and has become a high priority for the World Health Organization.^15^ Currently, however, there are no ETEC vaccines commercially available and there are numerous scientific challenges (e.g., heterogeneity of potential target antigens,^4^ poor mucosal immunogenicity responses, and potential safety issues of with antigens) as well as cost hurdles (e.g., develop, manufacture, and commercialize for use in the developing world) that impede ETEC vaccine development.^7^, ^13^

Owing to these challenges, there is growing interest in the use of passive immunization strategies to treat ETEC-induced diarrheal diseases in targeted populations by oral delivery of neutralizing immunoglobulins. For example, local delivery of antibodies that bind and neutralize ETEC in the GI tract could be used to prevent infection. Multiple virulence factors from ETEC have been recognized as potential antigens for passive immunity,^10^, ^16^ including secretion heat-labile enterotoxin (LT) that directly induces diarrhea by prompting solute retention and loss of water absorption in the intestinal lumen. LT is a heterohexameric A-B subunit toxin comprised of a catalytically active A-subunit and 5 B subunits.^16^ Subunit A has ADP-ribosylation activity, which covalently modifies the subunit of the GTP-binding protein (Gs), leading to the constitutive activation of adenylate cyclase and production of 3′,5′-cyclic AMP (cAMP).^17^ Consequently, continuous release of chloride and water into the intestinal lumen occurs causing watery diarrhea. The 5 B subunits mediate LT binding to glycolipid and glycoprotein receptors on host cells.^17^ Thus, antibody-induced neutralization of LT enzymatic activity and inhibition of adhesion could potentially be effective in controlling ETEC infection.

Secretory IgA (sIgA) antibodies are of particular interest for passive immunization during oral administration due to their natural abundance in secretions and mucosal surfaces.^18^ As the most prevalent immunoglobulin isotype in mucosal membranes, secretory IgAs (sIgAs) play crucial roles in protecting gut mucosal surfaces from pathogens and toxins.^19^, ^20^, ^21^ Secretory IgAs function to promote clearance of pathogens, maintenance of intestinal homeostasis, direct neutralization of bacterial virulence factors (e.g., enterotoxins), and modulation of proinflammatory responses.^19^, ^20^, ^21^, ^22^ Therefore, sIgA mAbs are a potential therapeutic platform for passive immunization by oral administration.^23^ Secretory IgA antibodies consist of dimeric IgG-like molecules, linked by a joining chain (J-chain), and complexed with a secretory component (SC) chain.^24^ The SC protein is acquired as the polymeric immunoglobulin receptor cleaves upon transport across epithelial cells into mucosal surfaces and secretions. Secretory IgA antibodies are inherently more resistant to proteolysis by digestive enzymes when compared to IgG in the gastrointestinal tract.^25^, ^26^

In this work, 3 anti-LT isotype variants (sIgA1, sIgA2, and IgG1) were expressed and purified from CHO cells in quantities of ∼5-10 mg. A series of physiochemical methods were developed (to accommodate limited availability of material) and used for preformulation characterization of anti-LT sIgA1, sIgA2, and IgG1 mAbs including evaluating various structural attributes (i.e., primary structure, post-translational modifications, size heterogeneity and aggregation, conformational stability, relative solubility, and antibody binding), and downselecting the key structural attributes of the sIgA mAbs to monitor during stability assessments. To this end, we examined the stability profile of the 3 anti-LT mAbs under conditions that mimic the gastric phase of oral delivery using simulated gastric fluids in a modified, scaled-down version of an *in vitro* gastric digestive model. These results are evaluated in terms of relative rank-ordering of the pharmaceutical stability of the 3 anti-LT mAbs from the point of view of future formulation development work to optimize both storage stability as well as stability during oral delivery.

## Materials and Methods

### Sample Preparation

The 3 anti–heat-labile toxin (LT) immunoglobulins (sIgA1, sIgA2, and IgG1) were expressed in CHO cells and purified by MassBiologics (University of Massachusetts Medical School, Boston, MA). The antibodies were prepared in 10 mM sodium phosphate, 150 mM NaCl, pH 7.2 (phosphate-buffered saline [PBS]) and stored at 2°C-8°C. Protein concentration was determined by enzyme-linked immunosorbent assay (ELISA) using known concentrations of sIgA1, sIgA2, or IgG1 as standards. When the protein concentration was determined by UV-visible spectroscopy, extinction coefficients were calculated based on the primary sequences^27^ as 1.49, 1.49, 1.64 mL mg^−1^ cm^−1^ for sIgA1, sIgA2, and IgG1, respectively.

### Sodium Dodecyl Sulfate Polyacrylamide Gel Electrophoresis

Twenty microliters of each 0.2 mg/mL Ig sample was mixed with or without 1 μL of PNGase F (New England BioLabs, Ipswich, MA) and incubated overnight at 37°C. Both deglycosylated and glycosylated Ig samples were reduced with 50 mM dithiothreitol (Invitrogen, Carlsbad, CA) at 70°C for 30 min. Reduced and nonreduced samples were then mixed with 4X LDS loading dye (Life Technologies, Grand Island, NY) containing 100 mM iodoacetamide (Life Technologies) and incubated at 100°C for 5 min. Samples were cooled to room temperature and separated by Sodium Dodecyl Sulfate Polyacrylamide Gel Electrophoresis (SDS-PAGE) using NuPAGE 10% Bis-tris gels (Life Technologies) and MOPS running buffer (Life Technologies) at 150 V for 75 min. Gels were stained with Coomassie Blue R-250 (Teknova, Hollister, CA) and destained with 40% methanol and 10% acetic acid. Gels were digitized using an AlphaImager (Protein Simple, Santa Clara, CA) gel imaging system.

### Size-Exclusion Chromatography

A Shimadzu Prominence ultrafast liquid chromatography HPLC system equipped with a diode array detector (with absorbance detection at 214 nm) was used. The system was equilibrated at 0.5 mL/min flow rate in 0.2 M sodium phosphate buffer at pH 6.8 for at least 2 h. Ten μL of each Ig (10 μg total protein) was injected and separated by a TOSOH TSKgel G4000SWXL column (8 μm particle size, 7.8 mm ID × 30 cm) for sIgA or a TOSOH TSK-Gel BioAssist G3SWxl column (5 μm size, 7.8 mm ID × 30 cm) for IgG1 with a corresponding guard column operated at ambient temperature (Tosoh Biosciences) using a 30-min run time. Gel filtration molecular weight standards (Bio-Rad, Hercules, CA) were injected before and after the Ig sample sets to ensure integrity of the column and HPLC system. Potential presence of larger aggregates was determined by running Ig samples with and without the size-exclusion chromatography (SEC) column (i.e., protein percentage recovery). Greater than 95% protein recovery was obtained for each of the 3 mAbs by SE-HPLC, indicating minimal loss of protein (e.g., larger aggregates) by using optimized SE-HPLC conditions for sIgA versus IgG1. Data were analyzed using LC-Solution software (Shimadzu, Kyoto, Japan).

### Sedimentation Velocity Analytical Ultracentrifugation

Sedimentation velocity analytical ultracentrifugation (SV-AUC) was performed using a Proteome Lab XL-I (Beckman Coulter, Brea, CA) analytical ultracentrifuge equipped with a scanning ultraviolet-visible optical system. Samples were diluted to 0.2 mg/mL in PBS pH 7.2 and transferred into Beckman charcoal-epon 2 sector cells with a 12 mm centerpiece and sapphire windows. All experiments were performed at 20°C after at least 1 h of equilibration after the rotor reached 20°C. SV-AUC was performed at a rotor speed of 40,000 RPM and with detection at 280 nm. The data were analyzed using Sedfit software (Dr. Peter Schuck, NIH). The partial specific volume was calculated using Sednterp software (Professor Thomas Laue, University of New Hampshire) based on the primary sequence. The buffer density and viscosity used in the analysis were also calculated using Sednterp based on the composition of the buffer. The density and viscosity of PBS buffer were calculated to be 1.0059 g/mL and 0.01021 Poise, respectively. A continuous c(s) distribution model was applied with a range from 0 to 15 Svedbergs, with a resolution of 300 points per distribution and a confidence level of 0.95. Baseline, radial-independent noise, and time-independent noise were fit parameters, while the meniscus and bottom positions were set manually.

### LC-MS Peptide Mapping

Ninety microliters of 0.5 mg/mL Ig samples were reduced with 3 μL of 0.5 M dithiothreitol for 30 min at 80°C and alkylated with 6 μL of 0.5 M iodoacetamide for 30 min at 37°C in the dark. The samples were then incubated overnight at 37°C with 12 μg of trypsin or chymotrypsin (∼1:25 enzyme:Ig ratio). The following day, the samples were heated to 98°C for 5 min to inactivate the enzyme. After cooling, samples were treated with PNGase F (New England BioLabs, Ipswich, MA) as described above to remove N-linked oligosaccharides. Before LC-MS injections, 0.05% (v/v) trifluoroacetic acid was added, and samples were centrifuged for 5 min at 14,000× *g*. The peptides from the digested protein solution were then separated by reversed phase UHPLC (Thermo Scientific) using a C18 column (1.7μm, 2.1 × 150 mm, Waters Corporation, Milford, MA) and a 85 min 0%-30% B gradient (A: H_2_O and 0.05% trifluoroacetic acid; B: ACN and 0.05% trifluoroacetic acid; 200 μL/min flow rate). MS was performed using an LTQ-XL ion trap (Thermo Scientific) and Xcalibur v2.0 software (Thermo Scientific). The instrument was tuned using a standard calibration peptide (Angiotensin II, Sigma) for maximal sensitivity before running any experiments. The mass spectra were acquired in the LTQ over a mass range of m/z 400-1900 using an ion selection threshold of 40,000 counts and a dynamic exclusion duration of 5 s. Raw experimental files were processed using PepFinder 2.0 software (Thermo Scientific). The database used for this experiment consisted of the primary sequences of all Ig molecules. Potential Cys carbamidomethylation, Asn deamidation, and Met oxidation were included in the analysis. Peptide assignments of tandem MS spectra were validated using a confidence score of >95%.

### Total Carbohydrate Analysis

A glycoprotein carbohydrate estimation kit (ThermoFisher #23260) was used to determine the total carbohydrate content (both N- and O-linked oligosaccharides) in Ig samples as a percentage of total protein mass. Before experiments, protein samples were buffer-exchanged into PBS at pH 7.2 using 30 kD MWCO filters (EMD Millipore, Billerica, MA) and a final concentration of 0.25 mg/mL Ig was used in each reaction. The recommended procedure provided by the manufacturer was used. Absorbance at 550 nm was determined using a SpectraMax M5 microtiter plate reader (Molecular Devices). Lysozyme, BSA, ovalbumin, Apo-transferrin, fetuin, and α1-acid were used as glycoprotein standards to construct a standard curve.

### N-Glycan Oligosaccharide Analysis

A GlycoWorks RapiFluor-MS N-Glycan Kit (Waters Corporation) was used to identify and quantify N-linked glycans following the manufacturer instructions. Briefly, Ig samples were centrifuged at 10,000 rpm for 5 min, 7.5 μL of 2 mg/mL Igs were mixed with 15.3 μL ultrapure water and 6 μL Rapi-surfactant and then heated at 90°C for 3 min. After cooling to ambient temperature, 1.2 μL of Rapi-PNGase F was added and samples were incubated at 50°C for 5 min to release N-linked glycans. Labeling was performed by combining oligosaccharide samples with 12 μL RapiFluor-MS reagent for 5 min. The reaction was diluted in 358 μL of acetonitrile and the reaction products purified using an HILIC μElution plate. The plate was washed 3 times in wash buffer (1% formic acid, 90% acetonitrile) and eluted in 90 μL of elution buffer. The samples were further diluted in 310 μL of diluent buffer. Fluor-MS N-glycan analysis was performed using an Agilent 1260 Infinity II HPLC system equipped with a 1260 FLD detector (Agilent, Santa Clara, CA) and an Agilent 6230 electrospray ionization time-of-flight mass spectrometer (Agilent, Santa Clara, CA). An HILIC AdvanceBio Glycan Mapping column (120 Å, 2.1 × 150 mm, 2.7 μm), that was operated at 45°C, was used to separate various N-glycans. Fifty microliters of prepared samples were injected into LC-MS system, with a flow rate of 0.6 mg/mL and a gradient run time of 55 min. Fluorescence data were obtained using excitation and emission wavelengths of 265 and 425 nm, respectively. MS was acquired simultaneously from 400 to 2000 m/z at a constant scan rate of one spectrum per second. N-glycans were assigned based on m/z values using a N-glycan database,^28^ and N-glycan quantification was calculated on integration of the fluorescence chromatogram.

### Conformational Stability Assessments

Thermal unfolding experiments were performed with Ig samples diluted in either PBS pH 7.2 or buffer exchanged into a modified simulated gastric fluid (SGF) containing 94 mM NaCl, 13 mM KCl with 10 mM citrate phosphate (CP) buffer at pH 3.0.^29^ Samples were then diluted in the corresponding buffer to a final concentration of 0.2 mg/mL. A fluorescence plate reader equipped with a charge-coupled device detector (Fluorescence Innovations, Minneapolis, MN) was used to obtain intrinsic tryptophan fluorescence spectra. Twenty microliters of each sample were loaded into a 384-well plate (Hard-Shell 384-well PCR plates) and overlaid with 2 μL of silicon oil (ThermoFisher Scientific, Waltham, MA). Samples were excited at 295 nm (>95% tryptophan emission) and the emission spectra were recorded from 300 to 450 nm with an integration time of 100 ms. Temperature ramps were programmed from 10°C to 100°C with an increment of 2.5°C per step. The mean center of spectra mass peak algorithm was used to analyze the data to determine the shift in fluorescence peak position as a function of temperature.^30^

Denaturant unfolding experiments were performed using 8 M stock solution of GdnHCl prepared in either PBS pH 7.2 or SGF containing 10 mM CP buffer at pH 3.0. Ig samples were buffer-exchanged and diluted to a final Ig concentration of 0.2 mg/mL, with a series of GdnHCl concentrations from 0 to 5.5 M. Ten microliters of each Ig sample was transferred to a 384-well plate (Hard-Shell 384-well PCR plates) and incubated at 4°C overnight before performing fluorescence measurements as outlined previously, but without the silicone oil overlay and at a fixed temperature (10°C). Data analysis was performed as described previously.^30^

### Relative Protein Solubility (Polyethylene Glycol Precipitation Assay)

Relative solubility of Igs was performed by adapting the method by Gibson et al.^31^ and Toprani et al.^32^ using smaller volumes. Briefly, 384-well polystyrene filter plates (Corning Life Sciences, Corning, NY) were used. Thirty percent of w/v PEG_10,000_ stock solutions were prepared in either PBS pH 7.2 or SGF containing 10 mM CP buffer pH 3.0. Various concentrations of PEG_10,000_ solutions ranging from 0% to 25% w/v were prepared with Ig concentration of 0.2 mg/mL for both buffer conditions. Samples were incubated overnight at room temperature in the dark. The next day, plates were centrifuged at 1233× *g* for 15 min and directly eluted into a clean 384-well plate. Relative protein concentration in each well was determined using a SpectraMax M5 plate reader (Molecular Devices) using detection at 214 nm. %PEG_midpt_ values were then calculated as described previously.^31^

### *In Vitro* Model of Gastric Digestion

To determine the stability of the proteins under simulated gastric conditions, each Ig was diluted in SGF, which was composed of 94 mM NaCl, 13 mM KCl, 0.15 mM CaCl_2_, along with 10 mM citrate-phosphate buffer pH 3.5 (added to maintain pH). Protein samples were either diluted directly into SGF-CP buffer alone or SGF-CP buffer with added bicarbonate buffer (9:1 ratio of the digestion solution and a bicarbonate neutralization buffer containing 0.03 M trisodium citrate and 0.3 M sodium bicarbonate at pH 8.5^33^) at a final protein concentration of 0.2 mg/mL. The reaction was started when the pepsin (2000 U/mL pepsin, Sigma)^29^ was added to the solution and samples were incubated at 37°C for varying amounts of time. The reaction was quenched by the addition of 400 mM NaOH to adjust to neutral pH. Samples were then analyzed by SDS-PAGE and ELISA. For ELISA analysis, samples were diluted in ELISA blocking buffer (0.1% BSA in PBS) at 40 μg/mL and 1 μg/mL of sIgAs and IgG1 digested samples based on the starting concentration, respectively, and stored at −20°C until analysis.

### Immobilized Pepsin Digestion

The Ig samples were diluted in SGF-CP buffer (see composition mentioned previously) at a final concentration of 0.2 mg/mL. Immobilized pepsin-agarose (ThermoFisher) was washed 3 times in SGF by centrifugation at 12,000× *g* before addition to the diluted Ig mAb mixture to a final pepsin concentration of 2000 U/mL. Samples were incubated at 37°C with end-over-end rotation to keep the beads in suspension. Beads were removed by centrifugation at 12,000× *g* for 1 min upon completion of the desired incubation times, and the supernatant was removed. Samples were then neutralized by addition of 400 mM NaOH before analysis by SE-HPLC. SE-HPLC was performed as described previously, but with an injection volume of 25 μL.

### Enzyme-Linked Immunosorbent Assay

In this work, 96-well affinity immunoassay plates (Thermal Scientific, Rochester, NY) were coated with 1.0 μg/mL heat-labile enterotoxin, B subunit (LTB) from *E. coli* (Sigma E8656) in PBS pH 7.2 and incubated overnight at 4°C. The next day, after removing coating solution, 96-well plates were filled with 200 μL of ELISA blocking buffer (0.1% BSA in PBS) for 2 h at room temperature. After flicking out blocking buffer, digested Ig samples were loaded 1:1 with blocking buffer and 1:1 serial dilutions were performed using blocking buffer as the diluent. Samples were incubated for 30 min at room temperature. Plates were washed 3 times with 0.05% Tween-20 in PBS, then 50 μL/well of HRP-conjugated Goat anti-human Ig protein (Fisher, Southern Biotechnology Associates) diluted in blocking buffer (1:15,000 was added and incubated for 30 min at room temperature. Plates were washed as before, and 100 μL/well TMB substrate solution (3,3′,5,5′-Tetramethylbenzidine [TMB]) was added and incubated for 5 min at room temperature in the dark. The reaction was quenched with 100 μL/well 1 M phosphoric acid. Optical density was recorded using a SpectraMax M5 microtiter plate reader (Molecular Devices) at 450 nm. Antibody binding to LTB by ELISA correlates with functional activity of the antibody as measured by GM1 holotoxin assay and Y-1 pathology assay (data not shown).

## Results

### Characterization of Purity, Primary Structure, and Post-translational Modifications of sIgA Versus IgG mAbs

**Figure 1:**
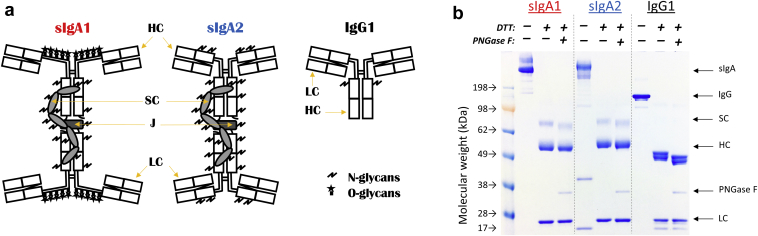
Structural overview and SDS-PAGE purity analysis of sIgA1, sIgA2, and IgG1 mAbs used in this study. (a) Schematic representation of immunoglobulin domains within sIgA1, sIgA2, and IgG1 antibodies. The LC, HC, SC, and J polypeptide chains, along with sites of post-translational N- and O-linked glycosylation, are indicated (see text). The sIgA1 and sIgA2 antibody species shown are commonly referred to as dimeric sIgA (see text). (b) Representative SDS-PAGE analysis of 3 anti-LT mAbs under nonreducing and reducing conditions with and without removal of N-glycans by PNGase F treatment. The molecular weight markers are on the far left lane. All bands assignments were based on molecular weight migration only.

In this work, we used various analytical tools to perform preformulation characterization of 3 anti-LT mAbs (sIgA1, sIgA2, and IgG1) to identify key structural attributes of the mAbs to then subsequently monitor for various stability assessments and for future formulation development work. As shown schematically in Figure 1a, sIgA antibodies are known to be composed of 2 IgG-like molecules which are disulfide linked by a ∼16 kDa joining chain (termed the J-chain), and also a ∼70 kDa SC chain that is complexed via the heavy chains in the Fc domains.^24^ The combined molecular weight of sIgA polypeptide chains is ∼380 kDa which increases to ∼435-460 kDa due to 15%-20% N-linked and O-linked glycosylation depending upon the sIgA subclass (see below). In comparison, IgG antibodies are less structurally complex and smaller (∼150 kDa with 1%-2% N-linked glycosylation). The sIgA antibodies shown schematically in Figure 1a are commonly referred to as dimeric sIgA in the literature^34^ (we use the terms sIgA and dimeric sIgA interchangeably in this work while we refer to the IgG antibody simply as IgG1). There are 2 main subclasses of sIgAs (sIgA1 and sIgA2) which structurally differ primarily in their hinge regions (e.g., length of hinge region, disulfide-bonding pattern, and the type and number of attached glycosylation sites)^35^ as depicted schematically in Figure 1a. Two and three conserved N-linked glycans are found on each Fc domain of sIgA1 and sIgA2, respectively, while sIgA2 also possesses 1 or 2 additional N-linked glycans on the C_H_1 domain. Another key structural difference is sIgA1 that contains multiple O-glycans on its elongated hinge region, while sIgA2 possesses a shorter hinge region that lacks such glycosylation.^36^ Both subclasses contain several N-linked glycosylation sites on the J-chain and SC.^37^

SDS-PAGE analysis was performed under nonreducing and reducing conditions, both with and without PNGase F treatment to remove N-glycans, as shown in Figure 1b. Under nonreduced conditions, both sIgA1 and sIgA2 mAbs displayed smear bands with a composition (based on migration within the gel vs. MW standards) of dimeric sIgA and higher molecular weight (HMW) species. The IgG1 sample migrated as primarily a single species at the expected MW of ∼150 kDa. For the sIgA1 and sIgA2 samples, the dimeric sIgA and HMW bands were shown to contain covalently cross-linked disulfide bonded species upon comparison to the reduced samples (consistent with previous reports).^38^, ^39^, ^40^ Under reducing conditions, 3 major components were identified for the sIgA mAb samples: the SC (∼70 kDa), heavy chain (∼50 kDa), and light chain (∼25 kDa). Although the J-chain (∼16 kDa) was not observed by SDS-PAGE (consistent with literature results; see Discussion), its presence was confirmed by LC-MS peptide mapping (see below). Specifically for sIgA2, we also observed 2 bands at relatively lower molecular weights (∼17 kDa and ∼40 kDa), which could represent a sIgA2 fragment (for the ∼17 kDa band), rather than J chain, due to their disappearance after reduction. The heavy and light chains of the PNGase F-treated reduced sIgAs migrated at slightly lower MW on the gel, indicating deglycosylation of these molecules. As expected, no migration differences were observed for the light chain bands independent of PNGase F treatment. By contrast, for the reduced IgG1 sample, both heavy (∼50 kDa) and light (∼25 kDa) chains were observed, and small amount of fragments (∼17 kDa) were also seen. Because the IgG1 heavy chain is N-glycosylated (see below), it also displayed lower MW migration after PNGase F treatment.

**Figure 2:**
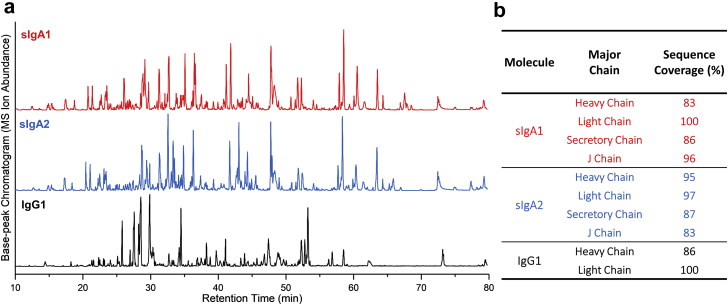
Representative LC-MS peptide mapping chromatograms of sIgA1, sIgA2, and IgG1 to confirm the primary sequence of each polypeptide chain and to evaluate for chemical post-translational modifications (see text). (a) Representative LC base peak chromatograms of each mAb after trypsin digestion; (b) percent primary sequence coverage for each polypeptide chain present in each of the 3 mAbs.

To confirm the primary sequence and identify potential post-translational modifications (PTMs), LC-MS peptide mapping was performed with the 3 anti-LT mAbs. On account of the requirement for PNGase F treatment for successful chromatographic resolution (data not shown), contributions of the N-glycans were not evaluated, and this PTM was examined separately (see below). On account of the sequence similarity (>97%) of sIgA1 and sIgA2, including both the variable and constant regions, many peptides were similar in terms of elution profile (Fig. 2a). At the same time, some differences in peptide elution profiles were also observed thus demonstrating a unique profile for each sIgA. For IgG1, the base peak chromatogram was significantly different when compared to the sIgAs, indicating a distinct digestion profile. Therefore, “fingerprint” chromatograms were obtained for each of the 3 mAbs. The sequence coverage obtained for each of the polypeptide chains is shown in Figure 2b. Overall, >85% coverage was obtained for each polypeptide chain for each mAbs. The light chain displayed the best coverage (97%-100%), the heavy chain coverage was from 83% to 97%, and SC and J chain displayed 86%-87% and 83%-96% sequence coverage, respectively. In terms of PTMs, no notable chemical modifications on sIgA1 and sIgA2 mAbs were observed. For IgG1, N-terminal pyroglutamic acid formation and C-terminal lysine residual truncation were identified in the heavy chain, which are commonly observed PTMs with IgG1 mAbs.^41^, ^42^

**Figure 3:**
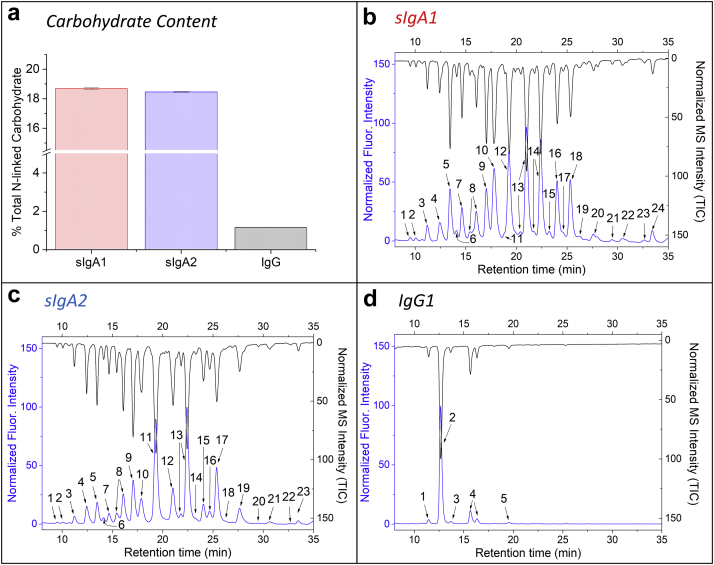
Glycosylation analysis of sIgA1, sIgA2, and IgG1 mAbs produced in CHO cells. (a) Total carbohydrate content, and (b, c, d) representative chromatographic profiles of Fluor-MS N-linked glycans removed from the mAbs are shown for (b) sIgA1, (c) sIgA2, and (d) IgG1. Fluorescence and mass spectrometry results are indicated, and peaks are numbered as a series of different N-glycans. See Figure 4 for summary of results. All data are presented as mean ± SD; *n* = 3.

**Figure 4:**
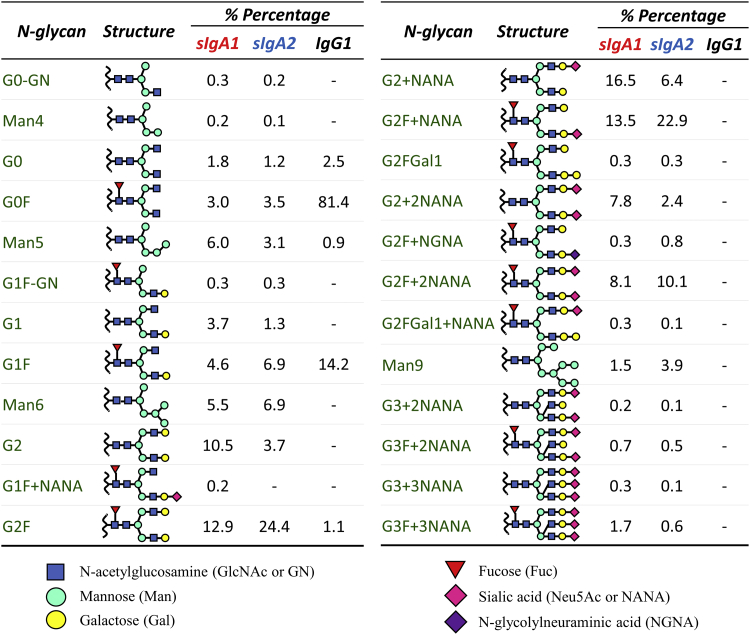
Identification and percent composition of each N-glycan type found in sIgA1, sIgA2, and IgG1 mAbs (produced in CHO cells) as determined by Fluor-MS N-linked glycan analysis. The total number of N-glycans, their respective oxford notations and structures,^47^ as well as the relative percentage of the total N-glycans for each mAb are shown. See Figure 3 for representative chromatograms. All data are presented as an average value; *n* = 3.

Glycosylation of antibodies plays an important role in functional activity (effector function and potentially antigen binding) as well as physical properties such as solubility and stability.^43^, ^44^, ^45^, ^46^ A combination of total carbohydrate content as well as the identification and quantification of the N-glycan oligosaccharide profile was determined for each of the 3 anti-LT mAbs. As shown in Figure 3a, a substantial difference in the total carbohydrate content between sIgAs mAbs (18.7% and 18.5% for sIgA1 and sIgA2, respectively), and IgG1 (1.2%) was observed. This result is consistent with the known structure and post-translational modifications of each mAb (Fig. 1a). Further analysis was performed to identify specific N-glycan type and relative quantification was performed by removal and derivatization of the N-glycans followed by chromatographic separation with detection by a combination of MS analysis and fluorescence measurements (see Methods). Twenty-four and twenty-three different N-glycans for sIgA1 (Fig. 3b) and sIgA2 (Fig. 3c) were identified, respectively, with G2+NANA and G2F + NANA observed to be the most dominant glycan types. By contrast, as shown in Figure 3d, the IgG1 mAb displayed a much simpler N-glycan profile, with 5 major N-glycan oligosaccharides, in which glycan G0F was the most dominant type (>80%). Each N-glycan type and corresponding percent composition found in each anti-LT mAb are summarized in Figure 4. It can be seen that the N-linked oligosaccharide composition and distribution greatly differs between the sIgA and IgG1 mAbs, as well as between the sIgA1 and sIgA2 mAbs.

### Characterization of Size and Aggregation Profile of sIgA Versus IgG mAbs

**Figure 5:**
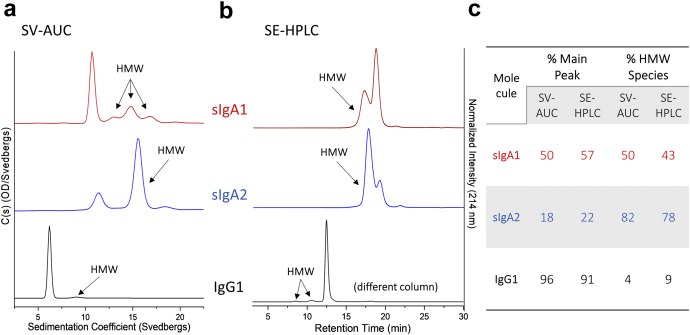
Size analysis of anti-LT sIgA1, sIgA2, and IgG1 mAbs as measured by SV-AVC and SE-HPLC. (a) Representative SV-AUC and (b) representative SE-HPLC analysis of the 3 mAbs. Two major categories (main peak and HMW species) were assigned based on sedimentation coefficient values and retention time values, respectively. Note, a different SEC column was used for IgG1 versus the 2 sIgAs mAbs. (c) Relative amount of main peak and HMW species calculated based on the total peak areas for both SV-AUC and SE-HPLC. Estimated molecular weight determinations were calculated as shown in Supplemental Table S1. Percent species were determined as average value; *n* = 2 for SV-AUC and *n* = 3 for SE-HPLC, with range and standard deviation values of 0.1% to 4.4% and from 0.1% to 0.6%, respectively.

In addition to size analysis under denaturing conditions by SDS-PAGE (see Fig. 1b), size distribution profiles under nondenaturing conditions were determined for each of the 3 anti-LT mAbs using 2 orthogonal methods, SV-AUC and SE-HPLC, as shown in Figures 5a and 5b, respectively. Two size categories (main peak and higher-order molecular weight species) were used to classify the size distribution of each sample. To be consistent with literature nomenclature, the main peak of the sIgAs is referred to as dimeric sIgA while the main peak for IgG is simply referred to as IgG1. Multiple species were identified by both SV-AUC and SE-HPLC, with SV-AUC displaying superior peak resolution between the main peak and the HMW species. Overall, similar percent composition results were observed in comparing SV-AUC and SE-HPLC size distribution results after peak area integration (Fig. 5c). Both sIgA1 and sIgA2 samples displayed a combination of main peak (dimeric sIgA) as well as relatively higher amounts of HMW species in solution (∼50% and ∼80% of total protein peak area, respectively, for sIgA1 and sIgA2 samples). For IgG1, a more homogeneous peak distribution was observed (it should be noted that to optimize separation of different species and percent recovery, different SEC columns were used for the sIgA vs. IgG1 samples, and thus the IgG1 eluted at an earlier retention time; see Methods) with <10% of total protein peak area in the form of HMW species. For each of the observed species, the molecular weight values were estimated, based on sedimentation coefficient values and comparison to the gel filtration standards for SV-AUC and SE-HPLC, respectively. As shown in Supplemental Table S1, the estimated molecular weight values of the main peak were calculated to be ∼430 kDa (with values ranging from 414 to 441 kDa) for the 2 sIgA mAbs and ∼150 kDa for the IgG1 mAb.

### Conformational Stability and Relative Solubility Assessment of sIgA Versus IgG mAbs

**Figure 6:**
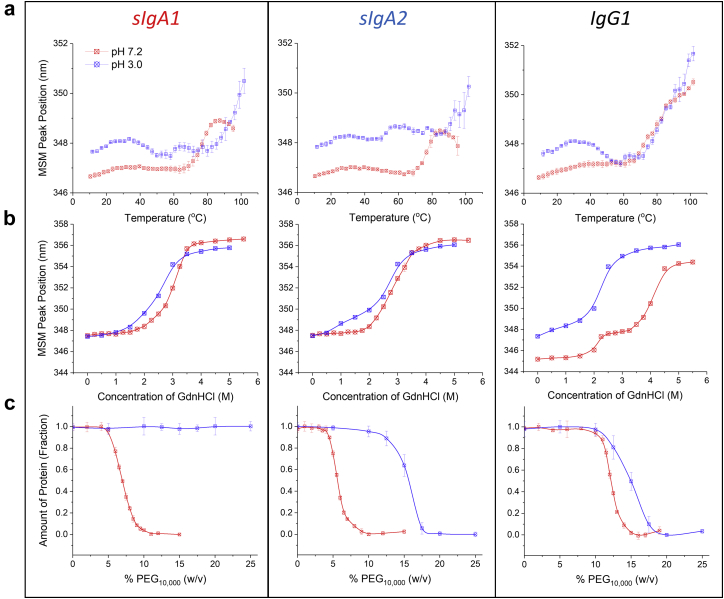
Comparison of conformational stability and relative apparent solubility profiles of anti-LT sIgA1, sIgA2, and IgG1 mAbs at pH 7.2 versus pH 3.0. (a) Thermal unfolding as measured by the shift of mean center of spectra mass peak position as a function of temperature, and (b) GdnHCl unfolding as a function of denaturant concentration, both measured by intrinsic Trp fluorescence spectroscopy. (c) Relative protein concentration of each mAb as a function of PEG_10,000_ concentration (w/v) as measured by UV-visible spectroscopy. All data are presented as mean ± SD; *n* = 3.

The sIgA1, sIgA2, and IgG1 mAbs were then compared in terms of their conformational stability and relative solubility profiles under 2 different pH solution conditions including pH 7.2 (to evaluate stability and relative solubility under storage conditions at neutral pH) and pH 3.0 (to evaluate stability and relative solubility under gastric conditions at acidic pH). First, the conformational stability of the mAbs was examined as a function of temperature. As shown in Figure 6a, solution pH effects the overall tertiary structure of the mAbs as a function of increasing temperature as determined by intrinsic Trp fluorescence spectroscopy. Each of the 3 mAbs has a similar lambda max value at 10°C and one major transition was observed to begin at 60°C-70°C at pH 7.2 for each of the mAbs. When the solution pH was decreased to 3.0, however, a red shift was observed at 10°C and multiple transitions were detected across the entire temperature range, and a red shift was observed at 10°C. These results suggest that the overall tertiary structure of each mAb is partially altered under acidic pH solution conditions and differences in their temperature melting profiles may be due to differences in partially altered structural states. Second, the conformational stability of the 3 mAbs was examined by the addition of increasing amounts of the chemical denaturant guanidine hydrochloride (GdnHCl), by monitoring changes in overall tertiary structure of the mAbs by fluorescence spectroscopy as shown in Figure 6b. The sIgA1 and sIgA2 mAbs showed one broad transition as the GdnHCl concentration was increased with a midpoint of ∼3M at pH 7.2. At pH 3.0, both sIgA mAbs showed lower conformational stability. Two distinct transitions were observed for IgG1 at pH 7.2, with midpoints of ∼2 and ∼4M GdnHCl. Lower conformational stability was also noted at pH 3.0 for the IgG1 mAb. Finally, in terms of pH effects on size and aggregation profiles, a preliminary SV-AUC experiment (*n* = 1) was performed to compare results at pH 7.2 (PBS buffer) to pH 3.0 (SGF without CP buffer), and no notable differences in the percent area of the major peaks (see Fig. 5) were noted for either the sIgA1, or sIgA2, or IgG mAbs (data not shown).

Interestingly, in terms of relative apparent solubility as measured by PEG-10,000 precipitation assay, a higher concentration of PEG-10,000 was required at pH 3.0 to precipitate each of the 3 anti-LT mAbs compared to pH 7.2 in the relative rank order of sIgA1 > sIgA2 > IgG1 (Fig. 6c). In fact, the sIgA1 remained soluble and failed to precipitate despite addition of the highest concentration PEG-10,000 (25%, w/v) when the solution pH was 3.0. Thus, higher relative apparent solubility was observed for each of 3 mAbs, albeit to various extents, by decreasing the solution pH from 7.2 to 3.0.

### Examination of sIgA Versus IgG Stability in an *In Vitro* Gastric Digestion Model to Mimic Oral Administration

To investigate and compare stability profiles of each of the mAbs under conditions that mimic oral delivery, we adapted an *in vitro* digestion model that focused on the gastric phase using simulated adult conditions for food digestion.^33^ In this adapted model, we scaled down the volume requirements and determined the most crucial experimental variables on mAb digestion rates including solution pH, digestion time, and pepsin concentration (data not shown). We fixed the solution pH to 3.5 (using a low concentration of 10 mM citrate phosphate buffer), optimized the pepsin concentration to 2000 U/mL, and monitored digestion in 1 mL solution as a function of time at 37°C (see Methods section). Three analytical techniques (ELISA, nonreducing SDS-PAGE, SE-HPLC) were used to assess the antigen binding activity, purity, and size of each of the anti-LT mAbs (and their degradation products) versus incubation time, respectively.

**Figure 7:**
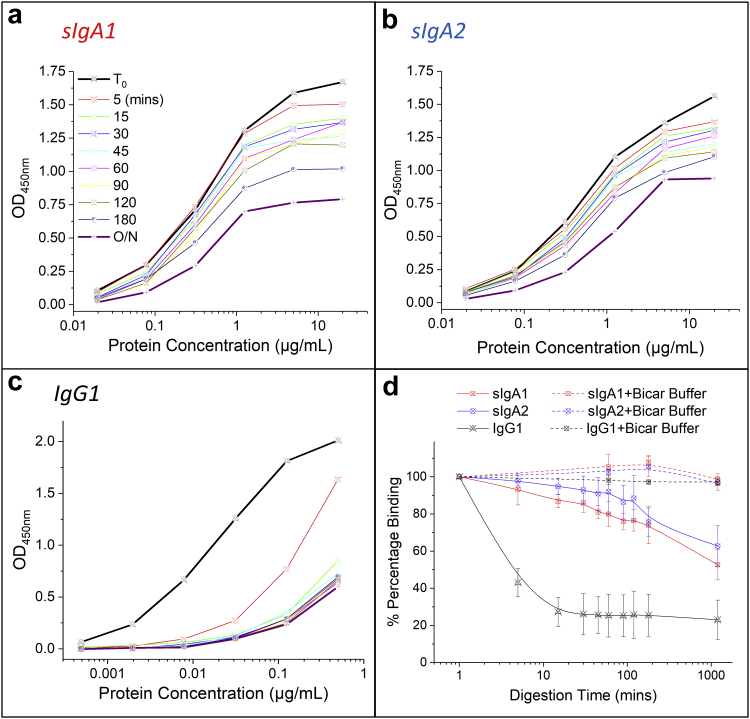
Comparison of LT antigen binding curves for the 3 anti-LT mAbs after incubation in the *in vitro* gastric digestion model as measured by ELISA including (a) sIgA1, (b) sIgA2, and (c) IgG1. (d) Comparison of the relative percent LT antigen binding remaining for each of the 3 mAbs based on normalization to the time zero binding curve (with or without bicarbonate buffer) is displayed (mean ± SD; *n* = 3).

First, the LT-antigen binding activity of each mAb was assessed by ELISA as a function of incubation time in the *in vitro* digestion model. The ELISA binding activity correlated well with antibody activities in functional assays including GM1 holotoxin assay and Y-1 pathology assay (data not shown). At time zero, the sIgA1, sIgA2, and IgG1 anti-LT mAbs bound the antigen in a concentration-dependent manner with a midpoint between 0.01 to 0.1 mg/mL mAb (Figs. 7a-7c, respectively). During incubation, a decreased signal (indicating decreasing amounts of mAb binding to the LT antigen) was observed for both sIgA1 and sIgA2. Nonetheless, no shift in the midpoint was noted and some antigen binding was still observed even after overnight digestion (Figs. 7A and 7B). By contrast, IgG1 lost its LT binding ability to a much greater extent (shift in the midpoint values as well as decreased total signal), and much more rapidly, when compared to the sIgAs (Fig. 7c). The majority of the binding capacity of IgG1 sample was lost after 5-10 min of digestion. To better compare these results across the 3 anti-LT mAbs, the percent loss of binding signal was calculated and the relative loss rates were then compared (Fig. 7d). For IgG1, the loss of mAb binding to LT antigen was rapid compared to the slower rates observed for both of the sIgAs. The coaddition of sodium bicarbonate buffer, which neutralizes the acidic pH leading to irreversible inactivation of pepsin,^48^, ^49^ resulted in ∼100% retention of LT binding even after overnight incubation in the *in vitro* gastric digestion model as shown in Figure 7d.

**Figure 8:**
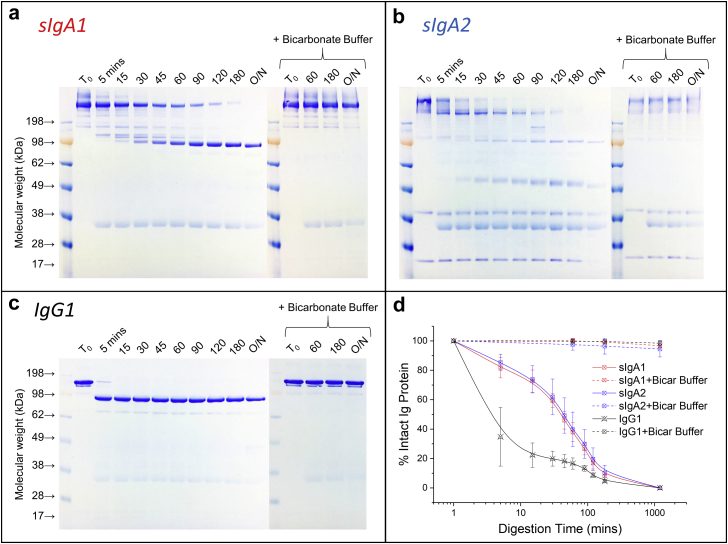
Comparison of structural integrity of the 3 anti-LT mAbs after incubation in the *in vitro* gastric digestion model as measured by SDS-PAGE: (a) sIgA1, (b) sIgA2, and (c) IgG1. Representative SDS-PAGE results of digested mAb samples over time with or without coaddition of sodium bicarbonate buffer (0.03 M trisodium citrate and 0.3 M sodium bicarbonate at pH 8.5). (d) Comparison of the relative percent intact species remaining over time for each of the 3 mAbs based on densitometry analysis of the main species (intact mAb) normalized to values at time zero (mean ± SD; *n* = 3).

Second, nonreducing SDS-PAGE was performed on the same sIgA1, sIgA2, and IgG1 samples incubated in the *in vitro* gastric digestion model (Fig. 8). The intact sIgA1 mAb (containing dimeric sIgA and HMW species as described previously) was gradually digested, and a series of digestion byproducts were observed including a major species ∼100 kDa, which presumably corresponds to the F(ab’)_2_ fragment (Fig. 8a). After 3 h, sIgA1 degraded mostly to the ∼100 kDa species. This result is consistent with known Fc susceptibility to pepsin digestion into smaller MW peptides while the more resistant F(ab’)_2_ fragment remains intact.^50^, ^51^ As expected, the intact sIgA1 species were essentially completely protected with coaddition of a sodium bicarbonate buffer (Fig. 8a). Overall, similar observations were made with the sIgA2 mAb as shown in Figure 8b; however, one difference was noted: in addition to the major digestion species of F(ab’)_2_, another protein species was detected at ∼50 kDa which was likely the Fab fragment. Addition of the bicarbonate buffer played a similar role in protecting sIgA2 from digestion by increasing solution pH. By contrast, IgG1 displayed an accelerated digestion profile when compared to the sIgAs (Fig. 8c). After the first time point (5 min), almost all the IgG1 was digested to F(ab’)_2_ fragments, and these remained after overnight incubation (Fig. 8c). The protective effect of bicarbonate buffer addition was also observed for IgG1. To more directly compare digestion profiles of the 3 mAbs by nonreduced SDS-PAGE, densitometry analysis of the native mAb band was performed (Fig. 8d). Although each of the intact mAbs were fully digested after overnight incubation (without addition of bicarbonate buffer), the rate of digestion of the sIgAs was much slower when compared to IgG1, indicating an increased resistance to acidic pH and pepsin digestion.

**Figure 9:**
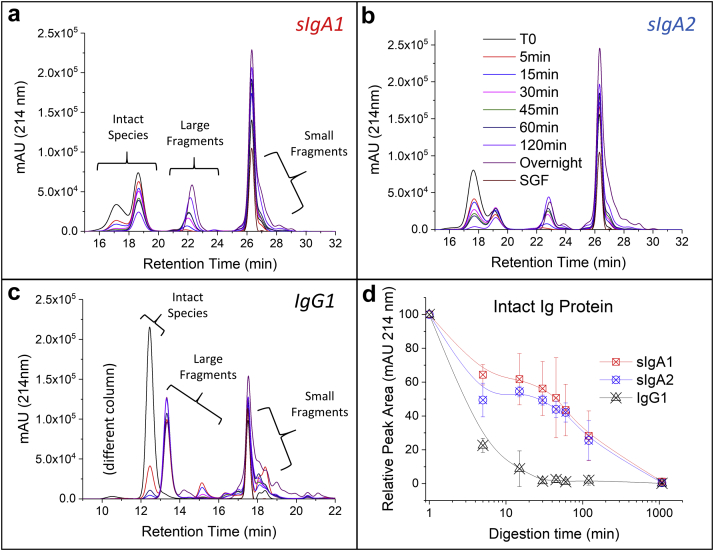
Comparison of the structural integrity of the 3 anti-LT mAbs during incubation in the *in vitro* gastric digestion model as measured by SE-HPLC: (a) sIgA1, (b) sIgA2, and (c) IgG1. Representative SE-HPLC chromatograms with UV 214 nm detection are displayed. Samples were incubated in presence of immobilized pepsin as a function of time and analyzed after removal of pepsin-agarose. (d) Comparison of the relative percent intact species remaining over time for each of the 3 mAbs based on main SEC peak integration and normalization to time zero (mean ± SD; *n* = 3).

Finally, SE-HPLC was also used to determine the size degradation profile of the 3 anti-LT mAbs (under nondenaturing conditions) by quantifying the decrease of the intact protein species and increase of the corresponding degradation products (Fig. 9). In this experiment, immobilized pepsin was utilized to easily remove the pepsin from the solution because pepsin coeluted with the sIgA degradation products in the SEC chromatograms (data not shown). Three major peaks were identified in the SEC chromatograms of the sIgA samples: the intact species (containing dimeric sIgA and HMW species as described previously), large fragments, and small fragments (Fig. 9a). Presumably, native protein is the intact species, while the F(ab’)_2_ and F(ab) are the large fragments and smaller peptide byproducts represent the small fragments. As shown in Figure 9a, digestion of intact sIgA1 was observed as a function of time where the main peak area decreased at each time point, while there was a concurrent increase in the large and small fragments. For sIgA2, similar trends were observed (Fig. 9b). For IgG1, 3 peaks were also observed; however, the digestion occurred more rapidly (when compared to the sIgAs) based on the reduction of the main peak area (Fig. 9c). To facilitate comparisons, the percent of intact mAb as a function of digestion time was determined. Both sIgA1 and sIgA2 demonstrated greater resistance to pepsin digestion when compared to IgG1, with no notable differences between sIgA1 and sIgA2 under these conditions (Fig. 9d).

### Comparisons of Stability and Solubility Profiles of Anti-LT sIgA1, sIgA2, and IgG Under Various Conditions

**Figure 10:**
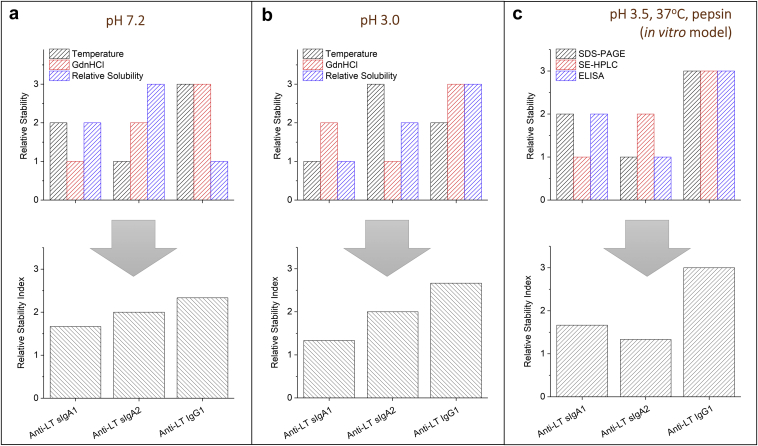
Comparison and rank ordering of desirable physical properties and stability profiles of sIgA1, sIgA2, and IgG1 mAbs (“relative stability indexes”; see text) including (a) physical properties at pH 7.2, (b) physical properties at pH 3.0, and (c) relative stability after incubation at 37°C, pH 3.5 with pepsin in the *in vitro* gastric digestion model. See Figure 6 and Supplemental Figure S1 for data sets used in (a) and (b). See Figure 7, Figure 8, Figure 9 for data sets used to rank order mAbs in (c).

To better summarize and compare the stability results described previously as a function of solution pH and molecule type (sIgA1, sIgA2, IgG1), a “relative stability index” was determined (Fig. 10). Briefly, Figure 10 displays the results of the relative stability comparisons between the 3 anti-LT mAbs in terms of conformational stability at pH 7.2 and 3.0 (vs. temperature and vs. GdnHCl) as shown in Figure 10a, the relative apparent solubility at pH 7.2 and 3.0 as shown in Figure 10b, and finally, the stability profile during incubation in the *in vitro* digestion model (37°C, pH 3.5 with pepsin) as shown in Figure 10c. For each condition, 3 values (1, 2, and 3) were assigned to each of the 3 mAbs corresponding to their relative rank ordering in stability (highest, intermediate, and lowest). These values in Figures 10a and 10b were determined from the replotting of Figure 6 data as shown in Supplemental Figure S1.

As shown in Figure 10a (top panel), the physical properties of the 3 mAbs at pH 7.2 were rank ordered as described previously, and then the results were combined (bottom panel). It can be seen that sIgA1 scored as having the best physical properties (combination of results from thermal and denaturant unfolding as well as relative solubility), followed by sIgA2 with intermediate behavior, and IgG1 as the least desirable properties overall. The same evaluation was carried out at pH 3.0 as shown in Figure 10b, and the same relative rank ordering of desirable physical properties was calculated at pH 3.0 with sIgA1 > sIgA2 > IgG1. Results of relative stability index during incubation in the *in vitro* gastric digestion model are shown in Figure 10c based on rank ordering the results from the ELISA, SDS-PAGE, and SEC analyses (see Figure 7, Figure 8, Figure 9). The sIgA1 and sIgA2 mAbs showed an overall similar ranking in terms of relative stability under conditions that mimic oral delivery, with IgG1 displaying the lowest stability overall under these conditions.

## Discussion

mAbs are widely used for treating a variety of diseases including preexposure prophylaxis (PreP) for infectious diseases, autoimmune disorders, and cancers.^52^ Approximately 70 mAbs are now approved for various therapeutic uses by regulatory agencies, and the vast majority are comprised of the IgG1 antibody subtype and are administered to patients by parenteral injection (intravenous or subcutaneous routes) for systemic delivery. From a product development point of view, key structural attributes of this category of antibody drugs (parenterally administered IgG1 mAbs) are now well established including determination of primary structure and post-translational modifications (e.g., glycosylation), size and aggregation propensity, higher-order structural integrity, and *in vitro* potency evaluations including antigen binding, cell-based assays, and in some cases, effector function assays.^53^, ^54^, ^55^ Once established, these structural attributes can be closely monitored during manufacturing, storage, and transport of an IgG1 mAb to ensure product quality.

### Analytical and Formulation Development Challenges for Orally Administered sIgA mAbs

In this work, we have evaluated some unique analytical and formulation development challenges with a different class of mAbs (secretory IgAs, sIgAs) for administration by a different route (oral administration for local delivery) for a different application (passive immunization to protect against enteric diseases in the developing world). Specifically, sIgA1, sIgA2, and IgG1 mAbs targeting heat LT, a major virulence factor of ETEC, were examined in this work. The potential therapeutic use of sIgAs for passive immunization is of particular interest because they are the predominant immunoglobulin isotype in tears, saliva, breast milk, colostrum, and mucosal surfaces such as the gastrointestinal as well as genitourinary tracts.^25^ Regardless of ultimate success of using anti-LT sIgA mAbs for passive immunization against ETEC infections *in vivo* (preclinical animal studies ongoing), generation of milligram quantities of these anti-LT sIgA mAbs provided the opportunity to evaluate sIgA mAbs in terms of pharmaceutical development challenges including preformulation characterization, stabilization, and formulation for oral delivery.

One key challenge for performing the preformulation characterization and stability evaluations reported in this work was the limited amount of purified sIgA material available. Given the current preclinical stage of development, only ∼5-10 mg of material was available for this work. To this end, we first evaluated a series of analytical tools to assess structural integrity, post-translational modifications, size and aggregation, conformational stability, relative solubility, and antigen binding activity with minimal material. In addition, we aimed to perform many of these assessments under conditions of neutral pH as well as more acidic pH (using a scaled-down version of an *in vitro* gastric digestion model; see below). The main objective was to not only better understand the key structural attributes of sIgA mAbs when formulated for oral administration but also compare the results to the much more widely studied IgG1 mAb. This was accomplished by examining 3 anti-LT mAbs produced in CHO cells (i.e., sIgA1, sIgA2, and IgG1).

### Key Structural Attributes of sIgAs as Potential Orally Delivered Drug Candidates

A combination of LC-MS peptide mapping, N-glycan analysis, and size comparisons under denaturing (SDS-PAGE) and nondenaturing conditions (SEC and SV-AUC) confirmed the increased structural complexity and heterogeneity of sIgA compared to IgG1 mAbs including multipolypeptide chain composition and higher molecular weight of the dimeric sIgA mAbs, as well as their more abundant and complex glycosylation patterns. In addition, a larger relative percent of HMW species was demonstrated for both the sIgA1 and sIgA2 mAbs versus the IgG1 mAb. The sIgA mAbs were more physically stable and pepsin-resistant (during incubation at 37°C, pH 3.5) and thus are likely more suitable for patient administration by oral delivery. This result is not unexpected considering sIgA as the most abundant antibody isotype in external secretions and mucosal membranes.^18^, ^56^ Based on these preformulation characterization results, 3 key structural attributes for sIgA mAbs were identified including (1) carbohydrate content including N-glycan oligosaccharide profiles, (2) size heterogeneity and aggregation, and (3) stability profile under *in vitro* conditions that mimic oral delivery as discussed in more detail below. The monitoring of these structural attributes during process development and scale-up geared toward lowering the cost of producing sIgAs (while maintaining product quality) will be of great importance to the overall success of this passive immunization approach.

When considering the total amount of carbohydrate and N-linked oligosaccharide profiles, significant differences were observed between sIgA1 versus sIgA2 (∼18% total carbohydrate with 23-24 different N-glycan oligosaccharides) versus IgG1 (∼1% total carbohydrate with 5 different N-glycan oligosaccharides) expressed in CHO cells. It is expected the glycosylation pattern for sIgA mAbs will be a critical structural attribute to monitor because their heavily glycosylated nature facilitates antibody binding to various pathogens and receptors.^37^ For example, the N-glycans on the J chain are usually required for dimer or oligomer formation of sIgAs and can also bind to polymeric immunoglobulin receptors (pIgR).^57^ The SCs of sIgAs are also heavily glycosylated, and the wide range of N-glycans on the SC creates diverse glycan epitopes, which can function as targets for lectins and bacterial adhesins.^37^, ^58^, ^59^ As a result, glycosylated SC can inhibit bacteria adhesion and prevent the establishment of an infection.^59^, ^60^ In addition, the galactose-terminating N-glycans are potential ligands for the asialoglycoprotein receptor that could mediate the clearance and half-life of IgAs.^61^, ^62^ Although a relatively simpler N-glycan profile was obtained for IgG1, these glycans are required for maintaining protein stability, increasing solubility, maintaining Fc effector functions, and receptor binding (e.g., Fcγ).^37^, ^63^ In terms of future work, analysis of the total carbohydrate content by mass spectrometry methods before sample manipulation will be of interest to determine. In addition, identification of the O-glycosylation profile for sIgA mAbs will need to be evaluated, both for process consistency as well as to better understand the role it may play in pathogen binding. Finally, batch-to-batch variability of the glycan profiles as well as their effects on sIgA mAb stability (in terms of overall flexibility of the hinge region and protection of the hinge region from protease digestion^37^) will be important topics to further study.

As for size heterogeneity of these 3 anti-LT mAbs, the IgG1 mAb was relatively more homogeneous containing 91%-96% main peak with smaller amounts of HMW species (4%-9%) as measured by SE-HPLC and SV-AUC. By contrast, both sIgA mAbs contained lower amounts of the main peak (dimeric sIgA at 50%-57% for sIgA1 and 18%-22% for sIgA2), and had higher levels of HMW species (43%-50% for sIgA1 and 78%-82% for sIgA2). Because there are several cysteine residues in each J-chain that usually form both interchain and intrachain disulfide bonds, it is likely that disulfide bond scrambling (leading to formation of interchain disulfide bonds between the tailpiece and cysteine residues in the heavy chains) can occur.^64^, ^65^ Thus, the J chain has the potential to be a hotspot for cross-linking oligomers for sIgAs.^64^, ^65^, ^66^, ^67^ In this work and consistent with published data, the J-chain was not detected by SDS-PAGE under reducing or nonreducing conditions,^68^ although it was readily identified by LC-MS peptide mapping. One possible reported explanation is that the J-chain remains associated with light chain of sIgAs as a complex and thus comigrated with the light chain,^68^, ^69^ although we did not observe such a complex by SDS-PAGE in terms of MW migration in this work (Fig. 1b).

It is expected that the presence and formation of HMW species observed in this work will be a key structural attribute to monitor in the future with various preparations of sIgAs. Aggregation is of concern with parenterally administered mAbs due to the loss of potency and the potential for anti-drug immune responses that limit efficacy and potentially affect safety.^70^ However, it is not known to what extent this would be a concern during oral delivery of sIgAs. In fact, the polymeric nature of sIgA may not necessarily be a negative attribute in terms of efficacy during oral delivery for passive immunization (as long as the mAb-based drug itself is not lost due to irreversible precipitation and no unwanted immune responses are generated). The biological potency of polymeric sIgAs has been previously reported to be preserved along with some protease resistance.^71^ Furthermore, polymeric sIgA may elicit intracellular signaling by binding to pIgRs and potentially inhibit intracellular virus replication.^72^, ^73^, ^74^ Interestingly, polymeric sIgA can display greater activity, when compared to dimeric sIgA, with regards to neutralizing toxins or whole bacterial cells, such as neutralizing proinflammatory antigens located in the apical recycling endosome.^64^, ^75^, ^76^ In addition, it has been recently revealed that the functionality of an sIgA against influenza A viruses is notably enhanced in a specific polymeric form (tetrameric) due to significant improvement of target breadth.^34^ Because it is likely that both covalent crosslinking as well as noncovalent interactions between sIgA molecules play a key role in formation of HMW species, future work will focus on better understanding of the mechanism(s) of aggregate formation during production and during long-term storage. The batch-to-batch variability of the percent content and size distribution of the HMW species for various sIgAs mAbs will also be of interest to further evaluate (both at time zero and during storage) as well as determining the effect of sIgA oligomerization on biological activity.

### Stability Profiles and Formulation Challenges of sIgA mAbs for Oral Delivery

The last key structural attribute identified in this work is the stability profile of sIgA1, sIgA2, and IgG1 mAbs under *in vitro* conditions that mimic oral delivery. We adapted a previously reported *in vitro* gastric model for evaluating the fate of various food products and supplements during transit through the digestive tract (see Methods).^29^ Owing to limited material availability, we focused our experiments on a scaled-down version of the gastric phase of digestion because this is the first major stage that is encountered *in vivo* with pepsin readily degrading proteins.^77^ As a preliminary formulation assessment, we also tested a bicarbonate formulation buffer, which has been successfully used as part of a rotavirus vaccination program during oral vaccination.^33^ The stability profile of the 3 anti-LT mAbs was monitored by ELISA, SDS-PAGE, and SEC. Although possessing a trend toward relatively lower binding affinity at time 0, sIgA mAbs showed greatly improved stability of antigen binding properties as measured by ELISA over 24 h incubation in the *in vitro* gastric digestion conditions (37°C, pH 3.5 in the presence of pepsin). The major digestion product after pepsin digestion was the F(ab’)_2_ fragment for each of the 3 mAbs as determined by SDS-PAGE and SE-HPLC. Nevertheless, the antigen binding values varied over time between the 3 mAbs. These observations indicate that not all F(ab’)_2_ fragments retained antigen binding activity to the same extent in comparison to the full-length, undigested mAbs. Potential conformational structure changes, allosteric effects, and glycosylation may influence these properties.^56^, ^78^, ^79^, ^80^, ^81^

In terms of future work with the scaled-down *in vitro* digestion model, sIgA stability profiles under conditions that mimic sequential digestion (e.g., oral, gastric, and intestinal phases) will be evaluated to better understand the stability profile of sIgA candidates under varying conditions that mimic the entire oral delivery pathway for local delivery to the GI tract. In addition, analytical techniques with better sensitivity and higher resolution, such as mass spectrometry, will be applied to assess the most sensitive sites for proteolytic digestion of the sIgA mAbs in the *in vitro* digestion model. In addition, this scaled-down *in vitro* digestion model can be used in the future to screen for formulation excipients that may help improve stability and retain potency during oral delivery. The concept was established in this work by demonstrating the protective effect of coaddition of bicarbonate buffer in terms of stabilizing the 3 anti-LT mAbs during incubation in the *in vitro* gastric digestion model (see Figure 7, Figure 8, Figure 9).

Smaller molecular weight protein therapeutic drugs (e.g., insulin) have been evaluated for systemic use by oral delivery^82^, ^83^, ^84^ and face several significant barriers including poor stability (due to acidic pH and digestive enzymes) and low bioavailability. It has been reported that advanced drug delivery systems can be used to improve oral delivery of insulin, for example, polymeric nanoparticles, micelles, liposomes, microspheres, or pH responsive complexation gels.^82^, ^84^, ^85^, ^86^ By contrast, the goal of this work is local delivery of the sIgA mAbs to bind and neutralize ETEC in the GI tract. Thus, passive immunization with sIgA mAbs may be a more successful approach than systematic delivery by the oral route of administration. However, for passive immunization applications in low-income and developing countries, low-cost formulations of sIgA mAbs for treatment of diarrheal diseases are critical, making the use of complex formulations such as advanced delivery technologies described previously less desirable.

To this end, future sIgA mAb formulation development efforts will focus on simple, low-cost liquid formulations that provide good long-term storage stability (ideally at room temperature), and concomitantly provide protection from acidic pH and proteases degradation during oral delivery, in a single final container. Ideally, such a low-cost liquid dosage form could be designed as an oral supplement during infant feeding. Based on results of this work, the commonly used PBS buffer formulation of sIgAs is not sufficient to meet these goals, so improved formulations will certainly need to be identified including optimization of solution pH and addition of excipients to minimize aggregate formation during processing as well as during long-term storage. In the shorter term, to facilitate first-in-human clinical studies of orally delivered sIgA mAbs in adults, stable refrigerated or frozen preparations of sIgAs, combined with bed-side mixing with additives to protect during oral delivery (for adults), can be considered. Thus, the analytical tools used in this work were selected for their ability to monitor key structural attributes of sIgA mAbs from a formulation development perspective. These tools can be used to not only ensure therapeutic sIgA mAb drug candidates are reproducibly produced but also can be formulated in a low-cost dosage form for oral administration, to pursue the long-term goal of protecting infants in the developing world against certain enteric diseases by targeted passive immunization.
