# Supplementary material for: Preformulation Characterization and Stability Assessments of Secretory IgA Monoclonal Antibodies as Potential Candidates for Passive Immunization by Oral Administration
Source: J Pharm Sci. 2020 Jan;109(1):407–21. doi: 10.1016/j.xphs.2019.07.018 (PMC6941217; doi:10.1016/j.xphs.2019.07.018)

**SUPPLEMENTAL INFORMATION**

**Preformulation Characterization and Stability Assessments of Secretory IgA Monoclonal Antibodies as Potential Candidates for Passive Immunization by Oral Administration**

Yue Hu^1,&^, Ozan S. Kumru^1^, Jian Xiong^1^, Lorena R. Antunez^1^, John Hickey^1^, Yang Wang^2^, Lisa Cavacini^2^, Mark Klempner^2^, Sangeeta B. Joshi^1^ and David B. Volkin^1*^

^1^Department of Pharmaceutical Chemistry, Vaccine Analytics and Formulation Center (VAFC), University of Kansas, Lawrence, Kansas, USA, 66047

^2^MassBiologics of the University of Massachusetts Medical School, Boston, Massachusetts, USA, 02126

*Corresponding Author: David B. Volkin, Multidisciplinary Research Building, 2030 Becker Dr., Lawrence, KS 66047. Phone: (785) 864-6262; Email: [volkin@ku.edu](mailto:volkin@ku.edu)

&Current Address: Bristol-Myers Squibb, 1 Squibb Dr, New Brunswick, NJ 08901

**Supplemental Figure S1.** Replotting of conformational stability and relative apparent solubility data sets for the three anti-LT mAbs in Figure 6 including intrinsic fluorescence peak emission maximum (MSM) values for each protein vs (A) temperature, and (B) GdnHCl concentration. (C) Relative mAb concentration in solution vs. amount of added PEG 10,000. Data sets compare and provide relative rank-ordering of the three mAbs at both pH 7.2 and 3.0 as shown in Figure 10. All data are presented as mean ± SD; n = 3.

**
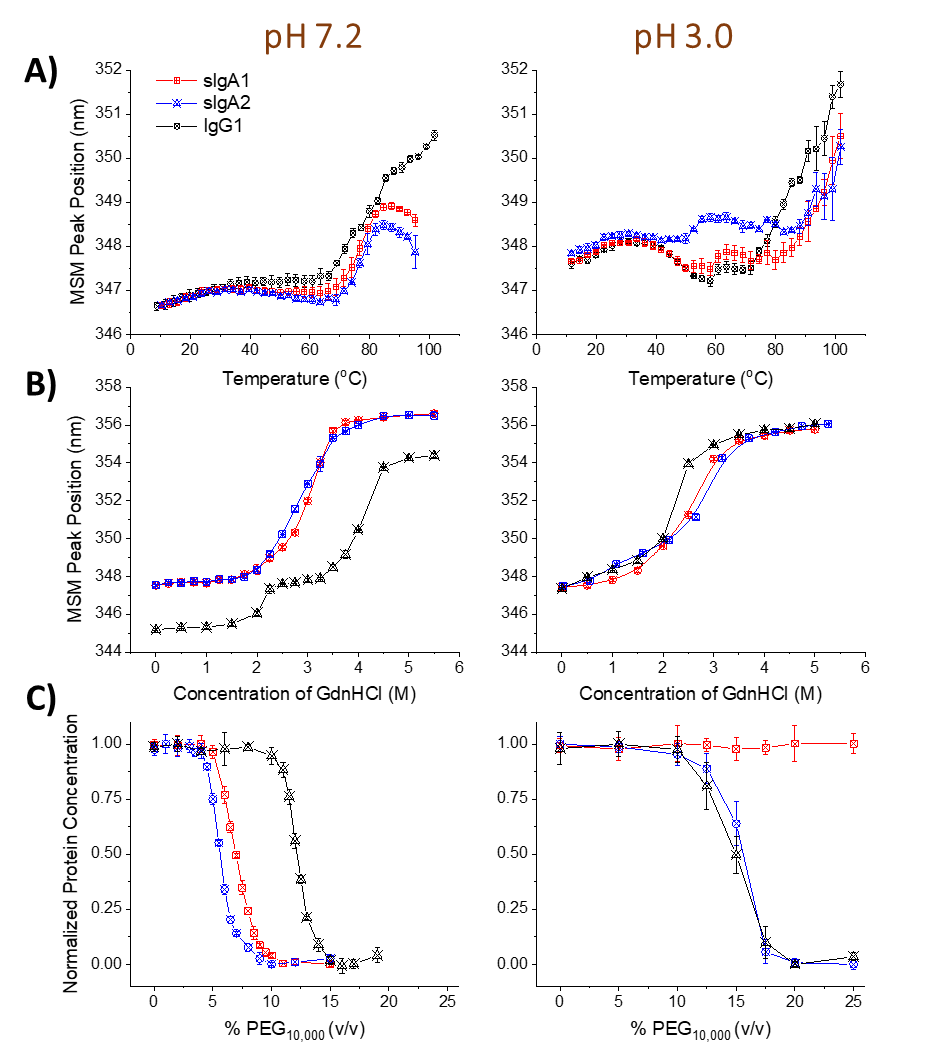
**

**Supplemental Table S1.** Estimation of molecular weight values of various species in solutions of the three anti-LT mAbs as determined from (A) sedimentation coefficient measurements from SV-AUC, and (B) retention time measurements from SE-HPLC (standard curve established using gel filtration molecular weight standards). Nomenclature of SV-AUC species (peaks 1-4) and SE-HPLC (peaks A-C) correspond to peaks from corresponding S values and retention times, respectively, as shown in Figure 5 (from left to right). Duplicate and triplicate measurements were performed for SV-AUC and SE-HPLC, respectively.


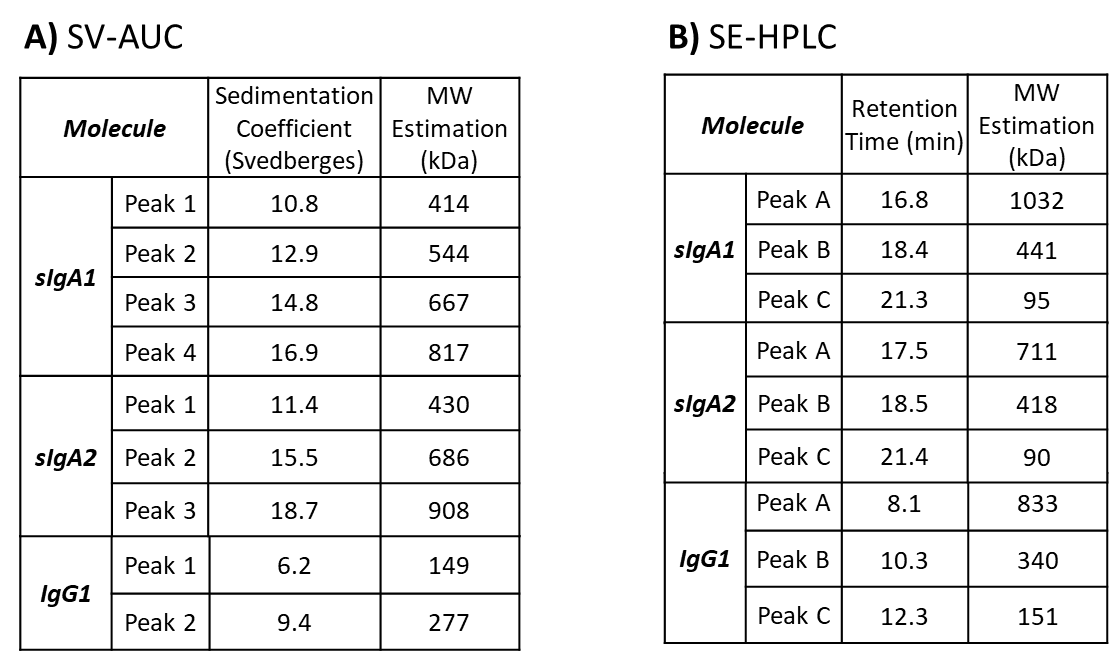

Supplement: Supplementary Information [file mmc1.docx]
